# A context analysis of bobbing and fin‐flicking in a small marine benthic fish

**DOI:** 10.1002/ece3.7116

**Published:** 2020-12-21

**Authors:** Matteo Santon, Felix Deiss, Pierre‐Paul Bitton, Nico K. Michiels

**Affiliations:** ^1^ Animal Evolutionary Ecology Department of Biology Faculty of Science Institute of Evolution and Ecology University of Tübingen Tübingen Germany; ^2^ Department of Psychology Memorial University of Newfoundland St. John's NL Canada

**Keywords:** blenny, predator detection, scorpionfish, signaling, triplefin, vigilance

## Abstract

Most antipredator strategies increase survival of individuals by signaling to predators, by reducing the chances of being recognized as prey, or by bewildering a predator's perception. In fish, bobbing and fin‐flicking are commonly considered as pursuit‐deterrent behaviors that signal a predator that it has been detected and thus lost its surprise‐attack advantage. Yet, very few studies assessed whether such behavioral traits are restricted to the visual presence of a predator. In this study, we used the yellow black‐headed triplefin *Tripterygion delaisi* to investigate the association between these behaviors and the visual exposure to (a) a black scorpionfish predator (*Scorpaena porcus*), (b) a stone of a size similar to that of *S. porcus*, (c) a conspecific, and (d) a harmless heterospecific combtooth blenny (*Parablennius sanguinolentus*). We used a laboratory‐controlled experiment with freshly caught fish designed to test for differences in visual cues only. Distance kept by the focal fish to each stimulus and frequency of bobbing and fin‐flicking were recorded. Triplefins kept greater distance from the stimulus compartment when a scorpionfish predator was visible. Bobbing was more frequent in the visual presence of a scorpionfish, but also shown toward the other stimuli. However, fin flicks were equally abundant across all stimuli. Both behaviors decreased in frequency over time suggesting that triplefin become gradually comfortable in a nonchanging new environment. We discuss why bobbing and fin‐flicking are not exclusive pursuit‐deterrent behaviors in this species, and propose additional nonexclusive functions such as enhancing depth perception by parallax motion (bobbing) or signaling vigilance (fin‐flicking).

## Graphical abstract

In fish, repeatedly and rapidly raising and lowering the anterior part of the body (bobbing) and repeated flicks of the dorsal, pectoral, or pelvic fins (fin‐flicking) are common behaviors often studied in the context of predation. We show that such behavioral traits can have other than an antipredator function in small marine triplefins. Improvement of depth perception by parallax motion by bobbing and signaling vigilance by fin‐flicking may represent additional, nonexclusive functions.

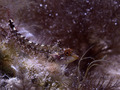

## 1 INTRODUCTION

Antipredator behaviors are ubiquitous and diverse in the animal kingdom. Alarm calls in birds, mammals, and fish (Ladich & Myrberg, 2006; Smith, 1965; Winn et al., 1964), along with avian mobbing behavior (Curio et al., 1978), stotting in cervids (Caro, 1986), schooling behavior in pelagic fish (Magurran, 1990), tail display in some birds, lizards, and mammals (Bitton & Doucet, 2013 for a list), and camouflage in moths (Kang et al., 2015), are among the topics that have received most attention. These behaviors reduce the risk of predation by alerting conspecifics of an imminent threat, by signaling a potential predator that it has been detected and thus reducing the chances of a successful capture attempt (pursuit‐deterrence), or by bewildering a predators’ visual system. Bobbing and fin‐flicking are two behaviors shown by fish in the visual presence of a predator, and often presumed to be pursuit‐deterrents (Brown et al., 1999; Karino et al., 2000; McCormick & Manassa, 2008; Murphy & Pitcher, 1997; Shennan et al., 1994).

Bobbing consists of repeatedly and rapidly raising and lowering the anterior part of the body. Nektobenthic species (e.g., Pomacentridae, Labridae) display bobbing while swimming (Ferrari et al., 2010). For example, tail‐spot wrasses (*Halichoeres melanurus*) bob their heads in response to the visual presence of predatory lizardfishes (Synodontidae) (Karino et al., 2000). In benthic fishes (e.g., Gobiidae, Blenniidae), bobbing is instead achieved by push‐ups with the pelvic or pectoral fins (Smith, 1989; Smith & Smith, 1989). For example, the starry goby (*Asterropteryx semipunctatus*) bobs in response to the visual presence of a predatory rock cod (*Cephalopholis boenak*) (McCormick & Manassa, 2008).

Fin flicks consist of repeated flicks of the dorsal, pectoral, or pelvic fins, often shown during investigative behavior. Glowlight tetras (*Hemigrammus erythrozonus*) fin‐flick to alert conspecifics and to signal a predator that it has been spotted (Brown et al., 1999). Fin flicks also signal the detection of a predator in European minnows (*Phoxinus phoxinus*) and convict cichlids (*Cichlasoma nigrofasciatum*) (Murphy & Pitcher, 1997; Shennan et al., 1994).

While most of the literature suggests that bobbing and fin‐flicking function as pursuit‐deterrents, it has rarely been tested if they are restricted to the visual presence of a predator, or if they are also used in the presence of nonthreatening conspecifics or heterospecifics while predators are not visible (Brandl & Bellwood, 2015; Cole & Ward, 1969; Ostrander & Ward, 1985; Tricas et al., 2006). Indeed, it has also been shown that fin flicks can be used for intraspecific communication (Smith & Smith, 1989). In rabbitfishes (Siganidae), fin flicks may be used as a signal by vigilant fish during coordinated feeding and can produce an acoustic signal (Brandl & Bellwood, 2015). In butterflyfish (*Chaetodon multicintus*), fin flicks produce single acoustics pulses that may function as agonistic displays between conspecifics (Tricas et al., 2006). In the orange chromide (*Etroplus maculatus*), fin flicks induce the formation of tight schooling behavior of young individuals around their parents in response to disturbance (Cole & Ward, 1969).

In this study, we investigated if the frequency of bobbing and fin‐flicking varies depending on the visual stimulus in a cryptobenthic species, the yellow black‐headed triplefin *Tripterygion delaisi,* while keeping other cues (chemical or sound) mixed. We focussed on manipulating visual cues only since in the natural environment triplefins are commonly exposed to mixed chemical and sound cues of predators, conspecifics and heterospecifics, which all share very similar ecological niches. This is further supported by the observation that bobs are not induced by smell in the starry goby, another cryptobenthic species which shares several ecological and life‐history traits with *T*. delaisi, such as being bottom‐dwelling, small, and cryptic (Brandl et al., 2018; McCormick & Manassa, 2008). Furthermore, previous laboratory and field experiments already showed that *T. delaisi* responds strongly to the visual presence of predators in comparison to control stones, while keeping chemical and sound cues mixed (Santon et al., 2020). We thus compared the response of triplefins to the visual presence of a cryptobenthic macropredator, the black scorpionfish (*Scorpaena porcus*), and to three harmless visual stimuli: a stone, a conspecific triplefin, and a nonpredatory cryptobenthic heterospecific combtooth blenny (*Parablennius sanguinolentus*). After confirming that *T. delaisi* shows avoidance reaction (keeps greater distance) toward its scorpionfish predator only, we compared the frequency of bobbing or fin‐flicking across stimuli with the expectation that they would occur the most in the visual presence of the predator.

## 2 MATERIALS AND METHODS

### 2.1 Model species

**FIGURE 1:**
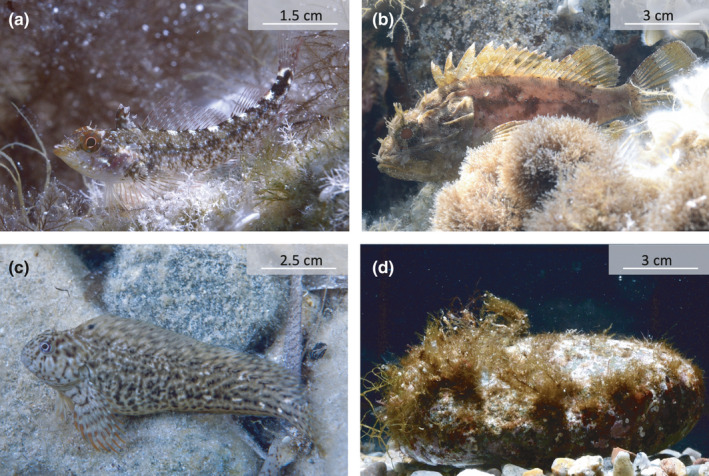
Examples of the four visual stimuli presented to triplefins. (a) The triplefin *T. delaisi*, focal species and conspecific stimulus in this experiment, (b) the scorpionfish *S. porcus*, (c) the blenny *P. sanguinolentus,* and (d) a natural stone of a size similar to that of the scorpionfish. All fish pictures were taken in their natural habitat in Corsica (STARESO, Calvi, Corsica, France). Picture credits: MS

*Tripterygion delaisi* (Tripterygiidae) is a small (standard length SL: 3–6 cm) cryptobenthic species found on rocky substrates from 5 to 30 m depth in the NE‐Atlantic Ocean and Mediterranean Sea (Figure 1a; Louisy, 2015). Except from breeding males, which display a yellow body with a black hood, individuals are highly cryptic. It mainly feeds on small invertebrates (Bitton et al., 2017; Fritsch et al., 2017; Michiels et al., 2018; Santon et al., 2019). This is an ideal species to investigate the function of bobbing and dorsal fin‐flicking because both are shown frequently, particularly when the fish is exploring an unfamiliar environment (Wirtz, 1978; Video S1). Triplefins are a special case with respect to fin flicks: Their dorsal fin is split into three parts (hence their family name). The first part seems to be used exclusively for fin‐flicking and shows a lot of variation in length and width between species and sexes (e.g., in the tropical genus *Enneapterygius*) (Fricke, 1997), perhaps suggesting an intraspecific communication function. Triplefins rarely swim in the open and have no specific hiding place as most blennies and small gobies do. Instead, they roam around cautiously on the substrate looking for microscopic prey while assessing their surroundings with high‐amplitude independent eye movement (Fritsch et al., 2017).

*Scorpaena porcus* (Scorpaenidae) is a cryptobenthic sit‐and‐wait macropredator (SL: 14–18 cm) from coastal marine hard substrates and seagrass habitats in the NE‐Atlantic and Mediterranean Sea (Figure 1b) (Louisy, 2015). Small cryptobenthic fish such as triplefins are often a component of its diet (Compaire et al., 2017; Santon et al., 2018).

*Parablennius sanguinolentus* (Blennidae) (SL: 8–12 cm) is a cryptobenthic species that is common in shallow rock pools in the NE‐Atlantic Ocean and Mediterranean Sea (Figure 1c; Louisy, 2015). This species feeds on algae. Its ecological niche marginally overlaps with the one of *T. delaisi*.

### 2.2 Study site, fish collection, and housing

We collected all specimens near the Station de Recherches Sous‐marines et Océanographiques (STARESO) located close to Calvi (coordinates: 42.58° N, 8.724° E), Corsica (France). Sampling took place under the general sampling permit of this station. All three species used for this study are nonthreatened and common. Individuals were caught using hand nets while scuba diving and transported in 50 ml perforated vials (triplefins and blennies) or in a custom‐made perforated box (scorpionfish). Once at the station, each species was housed separately in large tanks (L × W × H = 130 × 50 × 50 cm) illuminated by 150 W cold white LED floodlight (TIROLED Hallenleuchte, 150W, 16000 Lumen) equipped with a cyan filter (#172 Lagoon Blue, LEE Filters, Andover, England) to simulate light at depth. Lights were on from 07:00 to 21:00 hr. Rocks overgrown by algae provided natural structure. All aquaria received a permanent flow of fresh seawater. All fish were brought back to their natural sites immediately after the experimental trials were completed.

### 2.3 Experimental design

**FIGURE 2:**
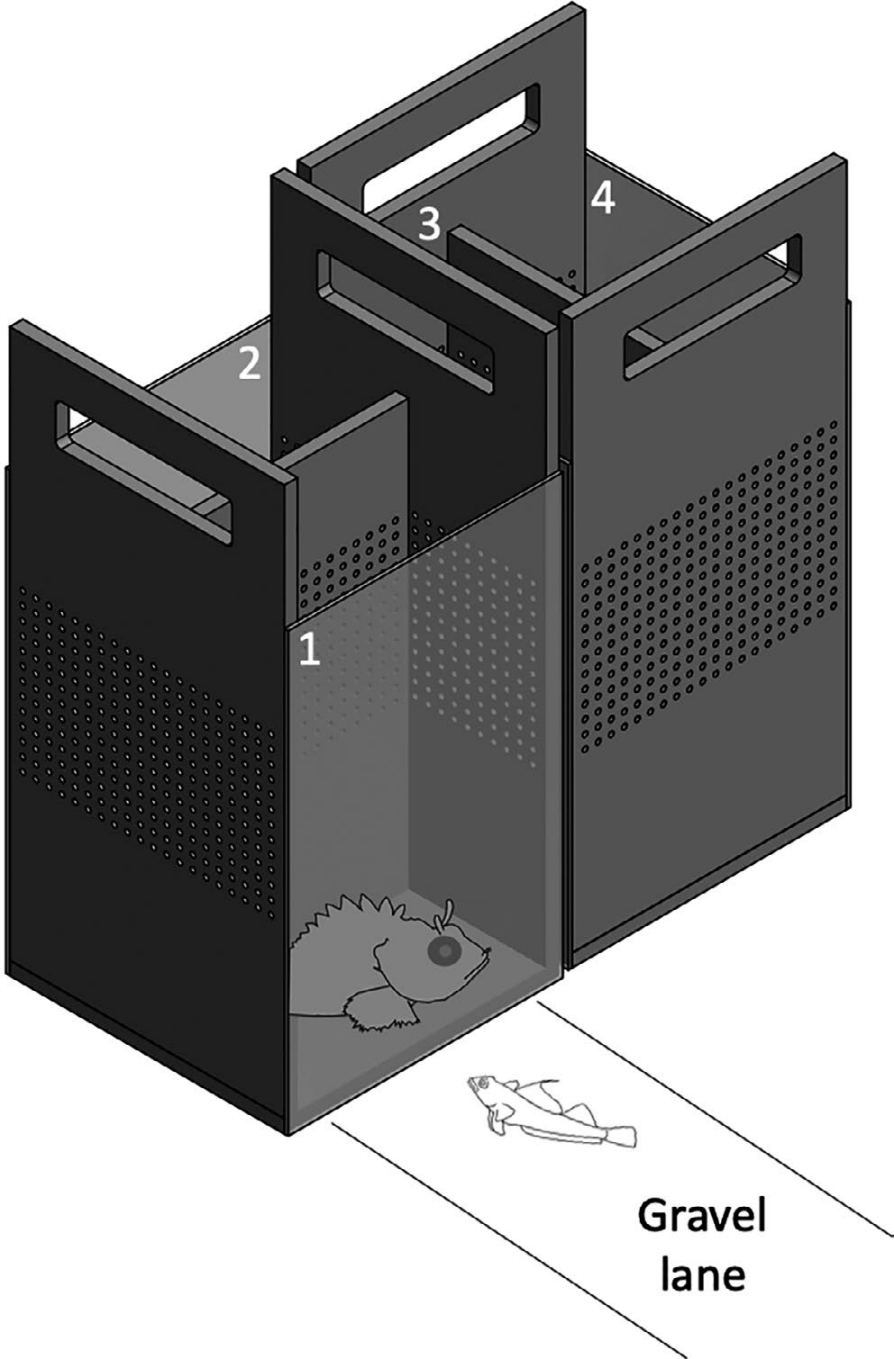
Two display twin boxes (L × W × H: 21 × 10 × 33 cm), each with two glass‐covered compartments used to alternate between stimuli. By rotating or swapping the boxes, only one stimulus could be made visible, while all were simultaneously present in the experimental tank. In this illustration, the triplefin is exposed to a scorpionfish in display compartment 1, while the other stimuli are hidden in compartments 2 to 4. The gravel lane encouraged triplefins to move along the observer's side of the aquarium, toward or away from a visual stimulus. Scheme by Gregor Schulte and MS

We tested 2 to 4 fish per day over 17 days in June 2018 between 09:00 and 12:00 hr in the morning, and between 14:00 and 17:00 hr in the afternoon in one aquarium (L × W × H = 110 × 50 × 40 cm) illuminated as described above. A white PVC plate that acted as a visual shield (L × H: 110 × 7 cm) was placed along the lower edge of the observer's side of the aquarium. It prevented the benthic triplefins from being distracted by outside movement in the room. The bottom of the aquarium was white and bare except for a strip of gravel (5 cm × 75 cm) along the observation side (Figure 2). Since triplefins prefer dark, structured substrates over bright, bare surfaces, the gravel lane encouraged them to move along the front window of the aquarium through which they were observed. A measurement scale placed on the side of the aquarium parallel to the gravel strip allowed to record fish distance from the visual stimulus with 0.5 cm resolution. As visual stimuli, we used 10 scorpionfish (standard length SL: 14–18 cm), 15 blennies (SL: 8–12 cm), 35 conspecific triplefins (one for each tested triplefin) (SL: 3–6 cm), and 10 stones similar in size to the scorpionfish (Figure 1d), all presented at one end of the gravel lane in a display box. The stimuli that were fewer than the number of triplefins tested were all used in random order before using them all again for subsequent trials. Although all four stimuli were simultaneously present in the tank, only one was visible to the focal triplefin in any given trial. We achieved this by placing the stimuli in perforated black PVC twin‐display boxes (L × W × H: 21 × 10 × 33 cm) with a clear front glass panel on opposite sides (Figure 2). In every box, each of the two compartments had a layer of small pebbles as substrate. Any stimulus could be made visible by turning the display window of its compartment toward the triplefin and placing it at the end of the gravel lane. The other stimuli were kept invisible by turning the display side away from the centre of the aquarium. The entire system was running under a permanent flow of fresh sea water. Therefore, all stimuli could be always smelled or heard, attributing any difference in response across stimuli to vision only.

Pilot trials allowed us to determine the optimal observation period, defined as the time window during which 90% of the triplefins responded to the stimulus. When released on the gravel lane, triplefins immediately started to show fin flicks and bobs while carefully approaching the display compartment. This is consistent with previous laboratory and field experiments that showed that a differentiated response between visual stimuli can be observed very soon after release in the experimental tank (Santon et al., 2020). Based on these tests, we set the observation time at 10 min for each stimulus. Each of 35 triplefins was exposed individually to all four stimuli in randomized order. Triplefins were gently released in the middle of the gravel lane and, during the subsequent 10 min, a single observer (FD) counted the number of bobs and fin flicks observed in one‐minute intervals and noted the distance from the tip of triplefins’ snout to the stimulus at the end of each full minute. Other typical behaviors in *T. delaisi* such as bright spots of focussed light on the iris, termed ocular sparks (Michiels et al., 2018; Santon et al., 2020), and tail flicks (Wirtz, 1978) were also noted, but ignored in the analysis due to their very low frequencies. Before each 10‐min session, the focal triplefin was temporarily placed in a dark container for 5 min while the visual stimulus was put in place. This process was repeated for all four stimuli for each focal triplefin.

### 2.4 Statistical analysis

Four focal triplefins did not move at all, or moved around the tank rather than staying on the gravel lane during the four trials. These individuals were ignored, leaving *n* = 31 triplefins for data analysis. We implemented a total of three models using the brms package for R v3.6.2 (Bürkner, 2017a, 2017b). This package fits Bayesian models using Stan, which is a C++ package for obtaining full Bayesian inference. In all three cases, we chose hurdle models to account for the zero inflation of the response variables median distance (27% zeros), number of bobs (50% zeros), and number of fin flicks (36% zeros).

We first tested if triplefins showed avoidance reaction toward the visual presence of the scorpionfish only, regardless of the mixed chemical and sound cues in the experimental tank. After visually assessing that distance was not strongly influenced by *observation time* (from minute 1 to 10), we implemented a hurdle gamma model (gamma distribution and log‐link for nonzero values, and Bernoulli distribution and logit‐link for zero values) on the median distance that triplefins kept from a stimulus for each 10‐min session. We modeled the non‐zero‐inflated and zero‐inflated part of the model using the main factorial predictor *stimulus* (scorpionfish, stone, triplefin, blenny).

Subsequently, to test if the frequency of bobs and fin flicks differed among the four visual stimuli, we implemented two hurdle negative binomial models (negative binomial distribution and log‐link for nonzero values, and Bernoulli distribution and logit‐link for zero values). Both models included the main factorial predictor *stimulus* (scorpionfish, stone, triplefin, blenny) and the main covariate *observation time* (from minute one to 10). The continuous covariate *observation time* was standardized (mean = 0, *SD* = 1) to facilitate model convergence. We modeled the zero‐inflated probability by *stimulus* only, as *observation time* did not seem to explain the presence of zeros. In both models, we also added a first‐order autoregressive (AR1) variance structure to correct for temporal dependency among the observations in each of the 10‐min sessions.

In all three models, the random component included *triplefin ID* (from 1 to 31) to account for the repeated measurements of each triplefin (Schielzeth & Forstmeier, 2008). We fit the models using weakly informative prior distributions (zero component: logistic(0,1) for intercepts, normal(mean = 0, scale = 5) for coefficients, nonzero component: student(*df* = 3, mean = −2, scale = 10) for intercepts, normal(‐3,5) for coefficients) and assessed overall model performance with posterior predictive model checking, that is, by comparing data predicted from the model with the observed data. For every model, we run 4 MCMC chains each with 8,000 iterations, which generated a total of 16,000 post‐warm‐up samples. Most model parameters showed reliable conversion indicators (Korner‐Nievergelt et al., 2015): an effective posterior sample size ≥10% of the total sample size, a Monte Carlo standard error ≤5% of the posterior standard deviation, and an $\hat{R}$statistic value ≤1.1 (Brooks & Gelman, 1998). We report effect sizes as the mean and 95% credible intervals (CI) of the model coefficients from the posterior distribution of the model parameters obtained with the MCMC simulation based on 16,000 post‐warm‐up samples. We also report the posterior probability of the hypothesis that the coefficients are greater than 0 to assess the importance of each predictor (explaining power substantial if such probability is almost 0 for negative coefficients or 1 for positive ones). We report and display graphically the median and 95% credible intervals of predicted response values for each of the four visual stimuli to assess pairwise differences. Such estimates were obtained by simulating 16,000 datasets, each computed using a different set of model parameters extracted from the posterior distribution. If the credible intervals of two groups do not overlap, the between group difference is substantial. All data were processed using R v3.6.2 (R Core Team, 2017).

## 3 RESULTS

### 3.1 Distance from each stimulus

**FIGURE 3:**
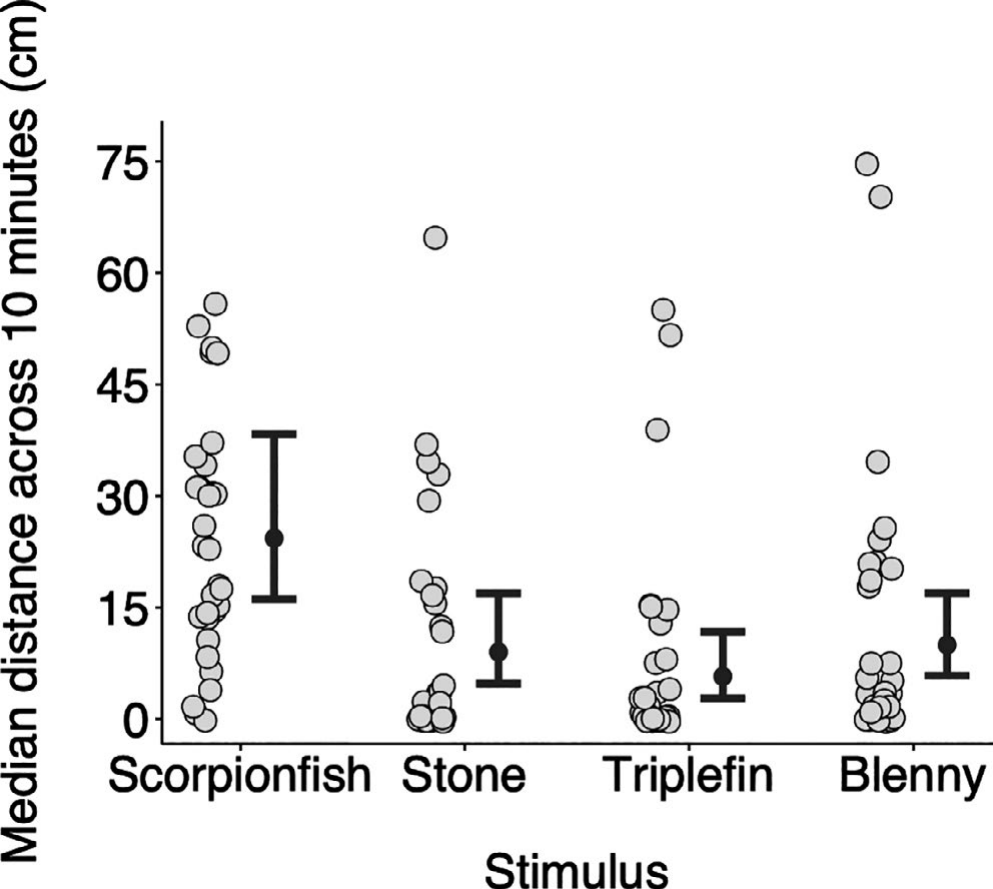
Median distance across 10 min as a function of stimulus. Triplefins stayed further away from the stimulus compartment when a scorpionfish predator was visible. Gray circles show median distances per individual (*n* = 31) across ten one‐minute intervals. Black dots and error bars represent predicted medians and their 95% credible intervals calculated from 16,000 simulations of the model (see Section 2). Pairwise differences can be visually assessed by the degree of overlap between the credible intervals of two groups. Symbols were jittered

The median distance triplefins kept from the display compartment differed across stimuli (Figure 3). Triplefins stayed further away when a scorpionfish was visible (median: 24.30 cm, CI: 16.08–38.34 cm) compared to a stone (median: 8.99 cm, CI: 4.73–16.87 cm), a conspecific triplefin (median: 5.72, CI: 2.78–11.73 cm) or a blenny (median: 9.94, CI: 5.82–16.89) (Figure 3, Table 1).

**TABLE 1:** Statistical analysis of the data presented in Figure 3

Predictors	Predicted mean	Lower CI	Upper CI	Prob. (coeff > 0)
(A) Median distance as a function of stimulus (n = 31, Bayesian R^2^ = .36)				
Nonzero values				
Intercept (scorpionfish)	3.24	2.83	3.69	1
Stimulus (stone)	−0.55	−1.20	0.09	0.04
Stimulus (triplefin)	−0.88	−1.55	−0.17	0.01
Stimulus (blenny)	−0.75	−1.35	−0.13	0.01
Zero values				
Intercept (scorpionfish)	−3.54	−5.62	−1.95	0
Stimulus (stone)	3.02	1.26	5.22	0.99
Stimulus (triplefin)	3.33	1.58	5.56	1
Stimulus (blenny)	1.90	0.06	4.12	0.98

Note

Hurdle gamma Bayesian model with median distance from the visual stimulus across 10 min as response variable. Predicted means and their 95% credible intervals (CI) are based on a gamma distribution with log‐link for nonzero values and on a Bernoulli distribution with logit‐link for zeros (see Section 2). For factorial predictors, estimates are computed using the indicated intercept level as reference. This choice is arbitrary and does not affect the overall conclusions. We also report (last column) the probability that the coefficient estimate is larger than 0. If this probability is almost 1 (for positive coefficients) or almost 0 (for negative coefficients), the predictor's contribution to the model is substantial (see Section 2).

### 3.2 Bobbing context

**FIGURE 4:**
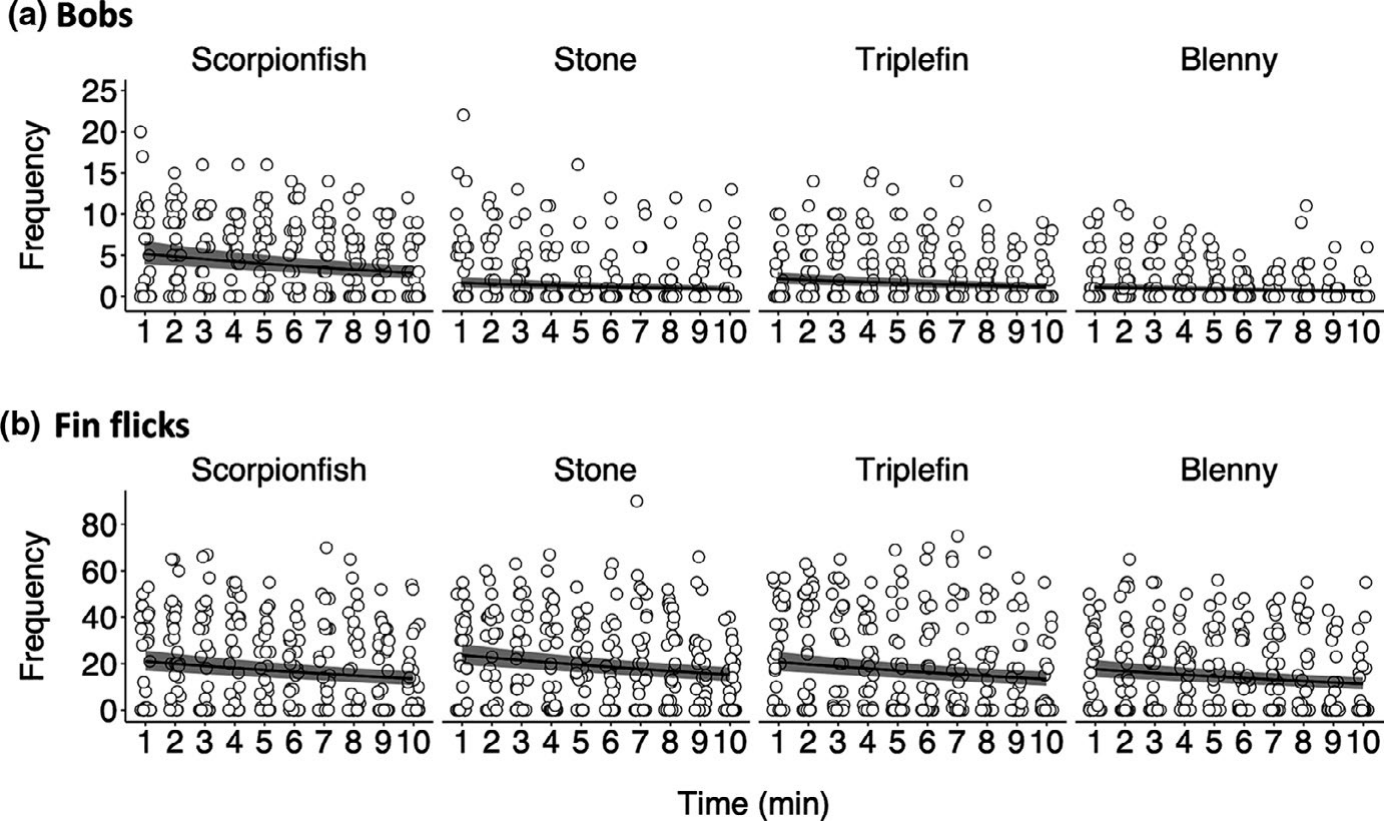
Number of bobs and fin flicks as a function of observation time and stimulus. Both behaviors become less frequent with increasing observation time. Data points show raw counts of bobbing (a) and fin flicks (b) for each individual (*n* = 31) in ten one‐minute time intervals. Lines and shades represent predicted medians and their 95% credible intervals on a continuous time scale calculated from 16,000 simulations of the model (see Section 2). Pairwise differences can be visually assessed by the degree of overlap between the credible intervals of two groups at the same observation time value (*x*‐value). Symbols were jittered

**FIGURE 5:**
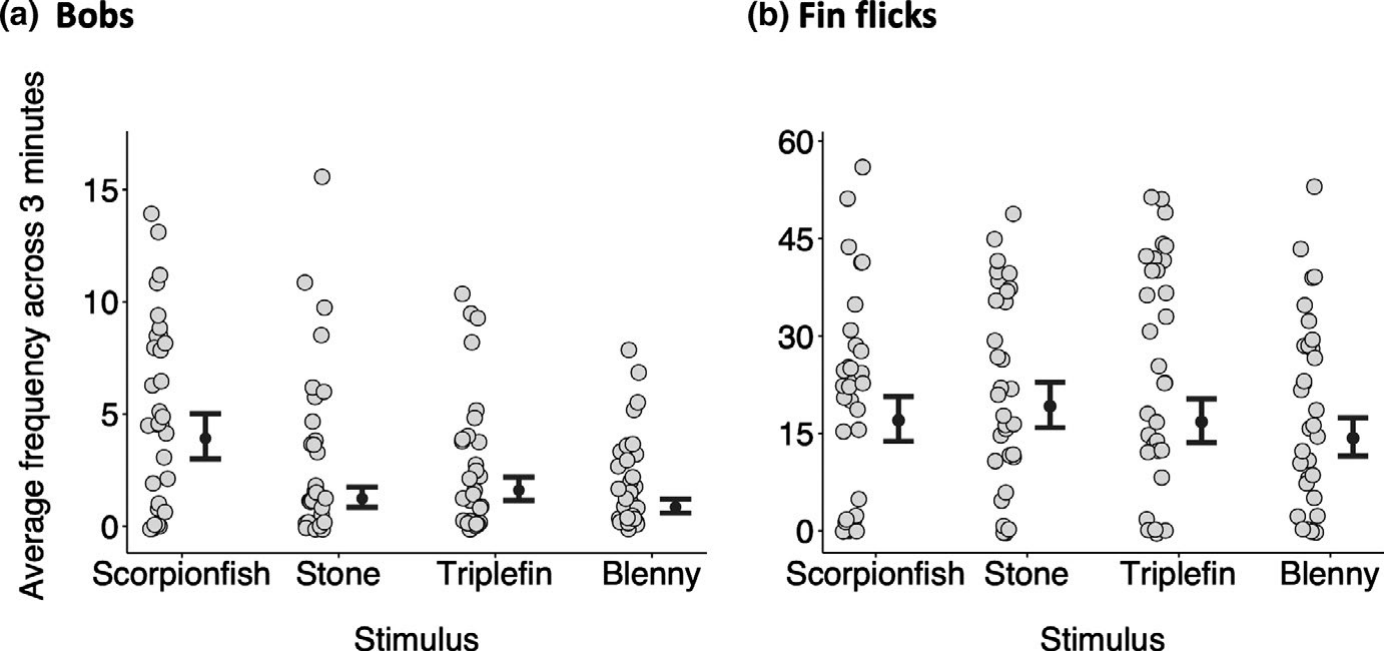
Average frequency of bobs (a) and fin flicks (b) across the first three minutes of the experiment as a function of stimulus. While mean bobbing frequency differed across stimuli (a), this was not the case for fin flicks (b). Gray circles show average numbers per individual (*n = 31*) across the first three one‐minute intervals. Black dots and error bars represent predicted medians and their 95% credible intervals calculated from 16,000 simulations of the model (see Section 2). Pairwise differences can be visually assessed by the degree of overlap between the credible intervals of two groups. Symbols were jittered

The absolute bobbing frequency differed across stimuli (Figure 4a). Triplefins showed more bobbing per minute when facing a scorpionfish (median: 3.91, CI: 3.00–5.01) compared to a stone (median: 1.24, CI: 0.85–1.75), a conspecific triplefin (median: 1.61, CI: 1.15–2.18) or a blenny (median: 0.86, CI: 0.58–1.22) (Figure 4a). Overall, bobbing frequency decreased linearly at a similar rate across stimuli with increasing observation time (Figure 4a, Table 2A). Concerning the absolute bobbing frequency, similar patterns can be observed also across the first three minutes of the experiment only (Figure 5a).

**TABLE 2:** Statistical analysis of the data presented in Figures 4 and 5

Predictors	Predicted mean	Lower CI	Upper CI	Prob. (coeff > 0)
(A) Bobbing frequency as a function of observation time and stimulus (n = 31, Bayesian R^2^ = .41, AR1 = 0.74)				
Nonzero values				
Intercept (scorpionfish)	1.73	1.53	1.93	1
Observation time (standardized)	−0.19	−0.26	−0.11	0
Stimulus (stone)	−0.60	−0.85	−0.35	0
Stimulus (triplefin)	−0.62	−0.86	−0.37	0
Stimulus (blenny)	−0.97	−1.23	−0.71	0
Zero values				
Intercept (scorpionfish)	−0.77	−1.21	−0.34	0
Stimulus (stone)	1.20	0.83	1.56	1
Stimulus (triplefin)	0.68	0.33	1.04	0.99
Stimulus (blenny)	1.20	0.84	1.56	1
(B) Fin‐flicking frequency as a function of observation time and stimulus (n = 31, Bayesian R^2^ = .15, AR1 = 0.74)				
Nonzero values				
Intercept (scorpionfish)	3.29	3.14	3.44	1
Observation time (standardized)	−0.14	−0.20	−0.08	0
Stimulus (stone)	0.01	−0.17	0.18	0.51
Stimulus (triplefin)	−0.06	−0.24	0.12	0.26
Stimulus (blenny)	−0.14	−0.32	0.04	0.07
Zero values				
Intercept (scorpionfish)	−0.53	−0.86	−0.19	0
Stimulus (stone)	−0.36	−0.70	−0.01	0.02
Stimulus (triplefin)	−0.12	−0.47	0.22	0.24
Stimulus (blenny)	0.10	−0.24	0.44	0.73

Note

Hurdle negative binomial Bayesian models with frequency of bobs (A) and fin flicks (B) shown every ten minutes of exposure to the visual stimulus as response variable. Predicted means and their 95% credible intervals (CI) are based on a negative binomial distribution with log‐link for nonzero values and on a Bernoulli distribution with logit‐link for zeros (see Section 2). For factorial predictors, estimates are computed using the indicated intercept level as reference. This choice is arbitrary and does not affect the overall conclusions. Both models include a first‐order autoregressive (AR1) variance structure to correct for temporal dependency among the observations in each 10‐min session. We also report (last column) the probability that the coefficient estimate is larger than 0. If this probability is almost 1 (for positive coefficients) or almost 0 (for negative coefficients), the predictor's contribution to the model is substantial (see Section 2).

### 3.3 Fin‐flicking context

The total number of fin flicks did not differ across stimuli (Figure 4b). Triplefins performed 16.99 (median, CI: 13.78–20.63) fin flicks per minute when facing a scorpionfish. This value was 19.17 (median, CI: 15.88–22.80) for a stone, 16.76 (median, CI: 13.57–20.26) for a conspecific triplefin, and 14.25 (median, CI: 11.49–17.36) for a blenny (Figure 4b). Overall, fin‐flicking decreased linearly at similar rates across stimuli with increasing observation time (Figure 4b, Table 2B). The total number of fin flicks still did not differ across the first three minutes of the interaction only (Figure 5b).

## 4 DISCUSSION

Triplefins kept a greater distance from the stimulus compartment only when a scorpionfish predator was visible. When visually exposed to the other three stimuli, triplefins approached the compartment more closely, despite the presence of chemical and sound cues of the predator. This result confirms the outcome of previous experiments that also showed a differential behavioral response of triplefins when visually exposed to either a predator or a natural stone in the presence of other than visual cues from a hidden scorpionfish predator (Santon et al., 2020).

In comparison to other studies (Brown et al., 1999; Karino et al., 2000; McCormick & Manassa, 2008; Murphy & Pitcher, 1997; Shennan et al., 1994), we found that bobbing and fin‐flicking were not related to a predator's visual presence only. Both behaviors are also shown when triplefins are visually exposed to natural stones, conspecifics, or heterospecifics.

Bobbing was significantly more frequent when a scorpionfish was visible compared to other visual stimuli. This difference further supports the idea that smell or sound alone trigger a weaker (if any) response than visual cues, as shown for another cryptobenthic marine fish, the starry goby (McCormick & Manassa, 2008). A possible explanation for the difference in frequency of bobs across visual stimuli is that bobbing is used as antipredator adaptation to signal the predator that it has been visually detected (Karino et al., 2000; McCormick & Manassa, 2008; Smith & Smith, 1989). This proposed pursuit‐deterrent function is ecologically relevant since a predator that relies on ambush predation such as a scorpionfish would not be able to surprise a prey that shows that it is aware of the predator's presence. However, the occurrence of bobbing also when the predator was not visible suggests that pursuit‐deterrence might not be the only function. Its role for communication between conspecifics seems weak since bobs also occur at similar frequencies in the presence of a stone or a blenny. As a possible alternative function, bobbing may improve vision. Moving the head up and down could enhance depth perception by motion parallax (Gibson et al., 1959; Rogers & Graham, 1979). Bobbing may therefore help discern objects that are hard to detect against their backgrounds (Kral, 2003). By moving the visual field, the target in the foreground exhibits faster and greater displacement than the background, thus improving 3D perception. This process has been studied in humans (Gibson et al., 1959; Rogers & Graham, 1979), and convincing evidence can be found for insects (Lehrer, 1994; Poteser et al., 1995; Wehner, 1994), birds (Frost, 1978; Van der Willigen et al., 2002), and mammals (Goodale et al., 1990; Legg & Lambert, 1990).

Additionally, triplefins may display occasional bobbing when exploring new potentially dangerous environments (e.g., where chemical smell of predators is present). In this context, low bobbing frequency could be used to display alertness before the threat has been visually detected. In our experiment, chemical cues such as predator smell could have induced some bobbing regardless of the stimulus shown, which then increased in frequency only when the predator was visible.

Contrary to other studies on a variety of species such as convict cichlids, rabbitfish, and butterflyfish (Brandl & Bellwood, 2015; Murphy & Pitcher, 1997; Shennan et al., 1994; Tricas et al., 2006), fin flicks were equally common for each stimulus. This suggests that at least in triplefins, they are neither a specific adaptation to signal the predator that it has been visually detected, nor a specific form of intraspecific signaling. Our experiment does not allow us to assess what function fin flicks fulfill. However, based on our own and Wirtz (1978) field observations, they may signal vigilance to "anyone," also to those observers who have not yet been visually detected but only sensed, for example, smelled or heard. This would explain why fin flicks were equally frequent across treatments, and fits well with the typical cautious exploratory nature of triplefin behavior. Additionally, fin‐flicking could also represent a subtle signal that induces curiosity and small movements in potential prey or predators, revealing their position to a triplefin.

Both bobs and fin flicks decreased in frequency across increasing time, suggesting that triplefins were getting more comfortable over time spent exploring a new nonchanging environment. Most Mediterranean cryptobenthic species such as blennies and gobies perform bobs and fin flicks, and occupy ecological niches similar to that of triplefins (Wirtz, 1978). Further investigations in related species might help clarifying why such behavioral traits evolved and specifically test the functions proposed by this study.

## 5 CONCLUSION

Our data show that a triplefin's antipredator response can be triggered by the visual presence of a threat. Cues other than visual are not sufficient to keep triplefins at a distance, neither to elicit high frequency of bobbing behavior. Yet, they might be a possible explanation for the occurrence of fin flicks, which could be used to signal alertness when a threat is sensed. In conclusion, we show that both bobbing and fin‐flicking can have more than a pursuit‐deterrence function in small marine cryptobenthic fish. Improvement of depth perception by parallax motion by bobbing and signaling vigilance by fin‐flicking may represent additional, nonexclusive functions.

## CONFLICT OF INTERESTS

We declare no competing interests.

## AUTHOR CONTRIBUTION

**Matteo Santon:** Conceptualization (equal); Formal analysis (lead); Software (lead); Supervision (equal); Visualization (lead); Writing‐original draft (equal); Writing‐review & editing (equal). **Felix Deiss:** Investigation (lead); Methodology (equal). **Pierre‐Paul Bitton:** Conceptualization (equal); Writing‐review & editing (equal). **Nico K. Michiels:** Conceptualization (equal); Project administration (lead); Supervision (equal); Writing‐original draft (equal); Writing‐review & editing (equal).

## Supporting information

The triplefin *T. delaisi* sporting bobs and fin flicks in its natural environment

## Data Availability

Raw data to reproduce the analyses can be found in Dryad (https://doi.org/10.5061/dryad.h18931zjn). R scripts available upon request.
